# Supplementary material for: MicroCT illuminates the unique morphology of Shiinoidae (Copepoda: Cyclopoida), an unusual group of fish parasites
Source: PeerJ. 2024 Mar 4;12:e16966. doi: 10.7717/peerj.16966 (PMC10921931; doi:10.7717/peerj.16966)
Supplement: Supplemental Information 2 [file peerj-12-16966-s002.docx]

| **specimen ID or USNM#** | **exposure timing (ms)** | **frame avg** | **skip** | **binning** | **sensitivity** | **number of images** | **voltage (kV)** | **current (uA)** | **power (W)** | **magnification** | **voxel size (um)** | **tube type** | **mode** | **target** | **collimator** | **focus** |
| --- | --- | --- | --- | --- | --- | --- | --- | --- | --- | --- | --- | --- | --- | --- | --- | --- |
| 1615601 (removed from 1659776) | 1000 | 3 | 1 | 1x1 | 2 | 1650 | 60 | 240 | 0.86 | 88.036 | 2.272 | 180 kv | 1 | diamond | y | 5 |
| 229112 | 1000 | 3 | 1 | 1x1 | 2 | 1350 | 70 | 360 | 0.77 | 100.6 | 1.987 | 180 kv | 2 | diamond | y | 3 |
| 229107 | 1000 | 3 | 1 | 1x1 | 2 | 1800 | 70 | 230 | 3.81 | 44.509 | 4.493 | 180 kv | 0 | diamond | y | na |
